# An Unusual Course of Metastatic Gastroesophageal Cancer

**DOI:** 10.1155/2015/941508

**Published:** 2015-12-03

**Authors:** William H. Smith, Sofya Pintova, Christopher J. DiMaio, Panagiotis Manolas, Dong-Seok Lee, Spiros P. Hiotis, Maria Kartsonis, Randall F. Holcombe, Kavita V. Dharmarajan

**Affiliations:** ^1^Icahn School of Medicine at Mount Sinai, New York, NY 10029, USA; ^2^Department of Medical Oncology, Icahn School of Medicine at Mount Sinai Hospital, New York, NY 10029, USA; ^3^Department of Gastroenterology, Icahn School of Medicine at Mount Sinai Hospital, New York, NY 10029, USA; ^4^Department of Surgery, Icahn School of Medicine at Mount Sinai Hospital, New York, NY 10029, USA; ^5^Department of Thoracic Surgery, Icahn School of Medicine at Mount Sinai Hospital, New York, NY 10029, USA; ^6^Department of Radiation Oncology, Icahn School of Medicine at Mount Sinai Hospital, New York, NY 10029, USA

## Abstract

We are reporting on a case of a 41-year-old woman who presented with metastatic gastroesophageal junction cancer and who achieved prolonged survival with a multimodal treatment approach. After initially experiencing robust response to chemotherapy, she was treated for distant recurrence with palliative radiation to the gastrohepatic and supraclavicular lymph nodes and subsequently, given her unusual near-complete response, with reirradiation to the abdomen with curative intent for residual disease. The case presented is unique due to the patient's atypical treatment course, including technically difficult reirradiation to the abdomen, and the resulting prolonged survival despite metastatic presentation.

## 1. Introduction

Cancers of the esophagus and gastroesophageal junction (GEJ) represent one of the most rapidly increasing types of tumor in many Western countries [1, 2]. In particular, the incidence of adenocarcinoma of the lower esophagus has risen dramatically in correlation to increases in the prevalence of known risk factors [1].

In localized or locally advanced disease resection has historically been considered the primary curative modality. Surgery alone has resulted in unsatisfactory survival outcomes, providing motivation for investigation of multimodal treatment approaches. Large randomized trials have demonstrated significantly improved survival with the addition of chemotherapy or chemoradiotherapy compared to surgery alone in early stage gastric and esophageal tumors [3–6].

Limited reports in the literature suggest that multimodality therapy may improve outcomes for some patients with advanced gastroesophageal malignancies [7]. Here, we report a case of GEJ adenocarcinoma that despite widespread disease at diagnosis achieved prolonged progression-free survival with minimal morbidity through an atypical combination of chemoradiation followed by reirradiation to the abdomen.

## 2. A Case Report

**Figure 1:**
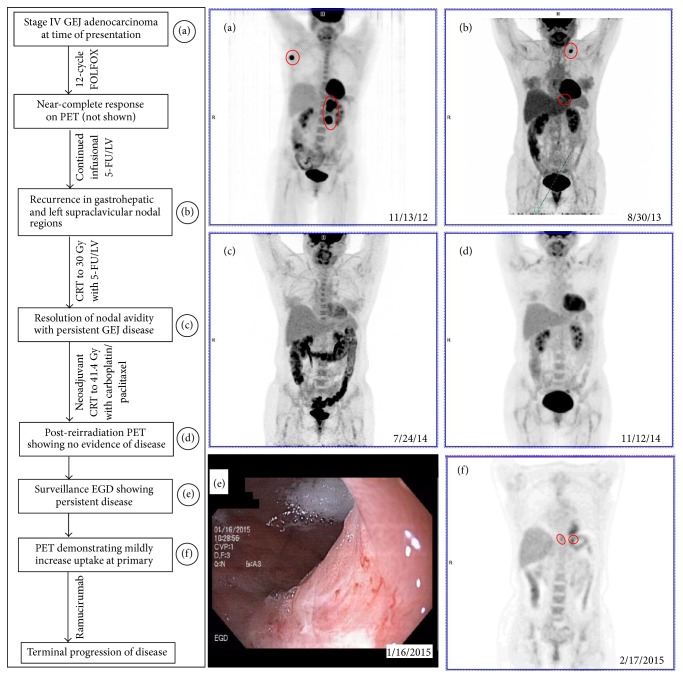
Selected PET/CT scans. (a) Initial PET/CT at the time of diagnosis showing uptake in GEJ, left para-aortic lymph nodes (SUV 11.8), and right axillary lymph nodes (SUV 10.8). (b) Surveillance PET/CT showing recurrence in the left supraclavicular fossa (SUV 23.3) and gastrohepatic nodal region (SUV 2.6). (c) PET/CT prior to second course of radiotherapy showing no residual metabolic uptake outside of the primary. (d) PET/CT post-reirradiation to the GEJ primary. (e) Shallow ulceration of GEJ with pathology demonstrating persistent adenocarcinoma. (f) Red circles highlight areas of increased uptake. Corresponding dates are shown.

In October 2012, a 41-year-old woman in otherwise excellent health presented with new onset abdominal and back pain to the emergency department at our institution where imaging revealed a solid gastrohepatic mass and associated left para-aortic lymphadenopathy. Endoscopic biopsy revealed invasive poorly differentiated HER2-negative adenocarcinoma of the GEJ. Histology demonstrated signet ring cells. Positron emission tomography (PET) demonstrated right axillary lymph node avidity (SUV (standardized uptake value) 10.8) and left para-aortic adenopathy (SUV 11.8) (Figure 1). Subsequent biopsy of the enlarged axillary lymph node confirmed the presence of stage IV adenocarcinoma of gastroesophageal origin. Peritoneal washings cytology was negative for malignant cells.

The patient began FOLFOX (5-fluorouracil, leucovorin, and oxaliplatin) chemotherapy. Restaging after 12 cycles of FOLFOX (oxaliplatin discontinued after cycle 10 secondary to neuropathy) demonstrated a near-complete response to therapy on PET scan. Patient continued on infusional 5-FU/LV. However, after additional 6 cycles of infusional 5-FU/LV surveillance, PET scan revealed recurrent disease in the left supraclavicular fossa (SUV 23.3) and gastrohepatic nodal region (SUV 2.6). The patient underwent a course of palliative radiotherapy to the two sites of recurrence at a dose of 30 Gy in ten once-daily fractions with concurrent 5-fluorouracil (FU) and leucovorin chemotherapy. The patient continued the infusional 5-FU/LV every 2 weeks after the completion of radiation for an additional 4 months. Follow-up PET 2 months following radiotherapy demonstrated resolution of avidity in both nodal regions and at the site of the primary tumor. However, endoscopy showed persistent, biopsy proven adenocarcinoma at the GEJ.

The case was then presented in our multidisciplinary tumor board. Given the patient's robust initial treatment response and excellent performance status, and in consideration of her prolonged survival up to this point despite metastatic presentation, we hoped that definitive treatment to the primary site would produce a favorable outcome.

Management options considered included palliative chemotherapy alone, surgical resection of residual disease, additional radiation to the GEJ with or without chemotherapy [5], or additional radiation to the GEJ with/without chemo followed by surgical resection [4]. After several conversations regarding the risks and benefits of the different treatment options, the patient elected to proceed with neoadjuvant chemoradiation (CRT) to the GEJ despite the potentially increased risks of acute and late toxicity associated with reirradiation to the abdomen.

**Figure 2:**
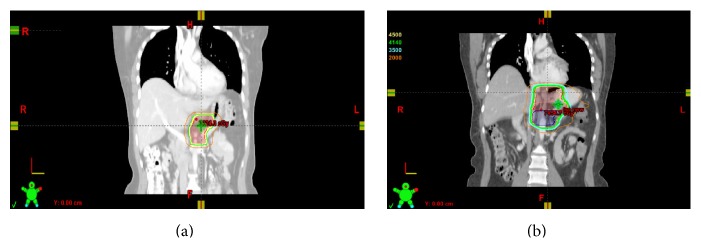
Palliative and reirradiation treatment to the abdomen. (a) Initial palliative radiation treatment to the gastrohepatic nodal region with corresponding isodose lines. (b) Reirradiation to the abdomen including the GEJ primary showing cumulative dose with corresponding isodose lines.

The patient thus completed a course of CRT to the GEJ primary to a dose of 41.4 Gy in 23 fractions with concurrent carboplatin (AUC = 2 × 5 weeks) and paclitaxel (50 mg/m^2^) per the CROSS regimen [4]. To increase the precision of the reirradiation treatment the patient underwent endoscopic ultrasound guided fiducial marker placement prior to simulation. The patient was placed in supine position and immobilized with a custom alpha cradle and compression belt and underwent CT simulation with 4D CT to document respiratory motion. The previously treated target volume was reproduced on the current planning scan to determine areas of overlap. A region of overlap including portions of the small bowel and stomach was limited to a cumulative dose constraint of 85 Gy in 2 Gy equivalents (Figure 2). Treatment was delivered with a 5-field IMRT plan to a planning target volume (PTV) that included the distal esophagus and proximal stomach up to the level of the fiducial markers. Daily cone beam CT imaging was used to minimize inaccuracies in setup and allow for a smaller than traditional margin around the PTV.

The patient tolerated treatment well with the exception of grade 3 nausea/vomiting, controlled with an escalated antiemetic regimen which included aprepitant, grade 2 neuropathy and grade 2 gastritis managed with sucralfate and proton-pump inhibitors. At follow-up visit she endorsed only mild treatment related grade 2 fatigue and grade 1 esophagitis. The patient decided to forego post-CRT resection for concern regarding the morbidity of the surgery and was instead continued on maintenance 5-FU/Leucovorin. PET at 2 months following completion of CRT showed no evidence of disease.

Surveillance EGD at 4 months following completion of definitive CRT showed persistence of adenocarcinoma at the GEJ primary. PET demonstrated mildly increased uptake at the primary site (SUV 3.2), suggestive of recurrence of local disease. The patient subsequently initiated treatment with ramucirumab. At 7 months following completion of CRT and 30 months following diagnosis, the patient was found to have progression of disease with interval development of peritoneal carcinomatosis. She developed complications of carcinomatosis with deterioration of her performance status. She was no longer a candidate for further anticancer treatment and enrolled in hospice. The patient passed away approximately 32 months after her initial presentation.

## 3. Discussion

GEJ cancer is a devastating disease that bears a dismal prognosis and commonly presents at an advanced stage, especially among younger patients such as the case presented in this report [8]. In metastatic cases, median survival is 6 months and 5-year survival is only 4% [8, 9]. Our patient lived 32 months after initial diagnosis of metastatic disease, much exceeding the median. We speculate that it may be in part due to the biology of her disease, site of her metastases (lymph node only at presentation), and multimodality therapy she received [10].

Management of patients with advanced, unresectable GEJ cancer represents a challenging scenario of continued uncertainty. External beam radiation therapy with concurrent chemotherapy is the standard approach for patients with locally advanced, unresectable disease. While this may provide sustained survival benefit in select locally advanced cases [11, 12], in metastatic disease such as in our patient the goal of care is most often palliative.

Treatment with fluoropyrimidine- or taxane-based chemotherapy is recommended in addition to supportive measures as first-line therapy in cases of metastatic GEJ cancer [13]. The role of FOLFOX chemotherapy in such cancers has been supported by multiple studies [14–17]. Nevertheless, no consensus yet exists regarding first-line chemotherapy regimen for metastatic disease. Our patient achieved an excellent response to FOLFOX and palliative CRT and was left without evidence of residual metastatic disease for 9 months.

When two surveillance studies suggested no other disease outside the primary site, the question became how to continue treating the patient. The patient was clear that she did not want to continue chemotherapy indefinitely due to the negative impact on her quality of life. We therefore hoped that treatment to the primary site with curative intent would be able to maximize her time off therapy. After an extensive discussion of the patient's case at the GI multidisciplinary tumor board, the management options under consideration included palliative chemotherapy alone, surgical resection of residual disease, additional radiation to the GEJ with or without chemotherapy [5], or additional radiation to the GEJ with/without chemo followed by surgical resection [4]. In locally advanced resectable disease, neoadjuvant CRT is the preferred treatment paradigm with definitive CRT reserved only for those patients who decline or are otherwise not fit to undergo surgery [13].

Surgery alone was not recommended in this patient, given the unacceptable risks of substantial morbidity and possible mortality in a patient who presented with metastatic disease. Moreover, even with pathologically complete resection, locoregional and distant failures are common. Several trials have demonstrated the benefit of neoadjuvant CRT versus surgery alone in GEJ cancer (Table 1) [3–5, 18–22]. However, radiation therapy would involve reirradiation of previously treated bowel, stomach, and esophageal tissues putting the patient at risk of perforation or fistula formation as some of these tissues had already received life-time tolerance doses of radiation.

The CALGB 9781/RTOG97-16 trial compared neoadjuvant CRT to surgery alone in patients with surgically resectable esophageal or GEJ cancer [5]. Median survival increased from 1.79 years in the surgery group to 4.48 years in the trimodality group (*p* = 0.002) [5]. In the setting of previous irradiation, dose used in the CALGB trial would not have been physically possible to achieve while maintaining dosing constraints.

The CROSS trial randomized patients with resectable esophageal or GEJ cancer to CRT (radiation to 41.4 Gy with concurrent carboplatin/paclitaxel) followed by surgery versus surgery alone [4]. An R0 resection was achieved in 92% of patients in the CRT arm compared to 69% in the control arm (*p* < 0.001), with 29% of adenocarcinomas showing a pathological complete response in the CRT arm [4]. Median overall survival was 49.4 months in the CRT arm compared to 24.0 months in the control arm (*p* = 0.003) [4]. This trial helped to establish the standard for treatment of locally advanced, resectable esophageal, and GEJ cancer [13]. While being technically difficult, reirradiation of the GE junctional region to a dose of 41.4 Gy would be feasible if carefully performed.

The literature on reirradiation to the abdomen is limited. Haque et al. first reported on a series of 13 patients who underwent reirradiation to the abdomen for gastrointestinal malignancies, finding that such treatment was generally well tolerated and provided a limited but clinically noteworthy duration of local control [23]. In this study, patients with a prior history of radiotherapy (median dose 45 Gy) were treated with a hyperfractionated course of 1.5 Gy fractions twice daily to a median dose of 30 Gy (range 24–48 Gy). Two patients terminated reirradiation early due to toxicity: one due to grade 3 abdominal pain and gastrojejunal anastomosis bleeding requiring hospitalization and one due to grade 2 duodenal ulceration and stricture [23]. Patients had limited overall survival (median survival 14 months), reflecting the poor prognosis of those with recurrent or metastatic abdominal malignancies included in this study [23]. However, only one patient in this cohort had gastric cancer (none had esophageal or GEJ cancer) and most received a lower retreatment dose following a longer retreatment interval than our patient [23].

A recent retrospective study of 10 patients who underwent reirradiation to the esophagus for recurrent esophageal squamous cell carcinoma demonstrated that such treatment is associated with a high risk for severe toxicity [24]. In this study, most patients (70%) experienced at least grade 2 toxicity (esophagitis in 4, dysphagia in 3, anemia in 1, and anorexia in 1) and 3 patients (30%) experienced esophageal perforation and tracheoesophageal fistula formation [24]. Those patients experiencing esophageal perforation and fistula formation received 50.4 Gy primary treatment followed by reirradiation to 45.0–50.4 Gy after an interval of 4.8–15 months [24]. Thus, while reirradiation was associated with a high risk for severe toxicity, relatively high cumulative doses were administered in these cases with a short interval between initial and retreatment.

After much consideration it was felt that for our patient the risks of surgery alone outweighed the potential benefits given the high likelihood of recurrence. Due to the patient's prior palliative radiation to the gastrohepatic nodal region we could not safely deliver the standard curative dose per CALGB 9781/RTOG97-16 (50.4 Gy) without overdosing the nearby bowel [5] and risking potentially unacceptable acute and late toxicities including fistulas and bowel obstructions. The patient thus underwent neoadjuvant CRT per the CROSS regimen because the lower radiation dose (41.4 Gy) would allow us to meet our dosing constraints for normal tissues while still treating with curative intent [4, 5]. This was contingent upon the patient planning for post-CRT resection to remove the primary tumor site and also the reirradiated tissues, thereby minimizing radiation-related toxicities.

The patient's PET showed no evidence of disease at 2 month following completion of CRT. In the CROSS trial, 29% of patients showed complete pathological response on resection [4]. Thus, despite this patient's ensuing locoregional failure, her initial response gave reason to suspect that CRT alone with 41.4 Gy dose of radiotherapy might be sufficient for cure. While cure was not achieved, this patient tolerated treatment relatively well and was still alive without progression of disease until 7 months following reirradiation.

There is no consensus treatment for recurrent GEJ adenocarcinoma. Our patient's case demonstrates that reirradiation to the abdomen may be safe and beneficial in select individuals. As the CROSS trial has helped define initial management of locally advanced resectable GEJ adenocarcinoma, most patients presenting with recurrence will have already undergone initial radiation to 41.4 Gy. In such cases, reirradiation to 30 Gy with concurrent chemotherapy would result in the same cumulative dose as in our patient and appears to be reasonable in select patients with good performance status. Advancing technological capabilities to provide more precise radiation delivery fields may improve our ability to treat recurrent GEJ cancers with reirradiation in the future. As such, we propose that while each patient must be considered in the context of his or her particular circumstances, reirradiation with concurrent chemotherapy should represent a preferred option for salvage therapy. Given the complexities of such treatment, a better understanding of the factors determining who would benefit most from this therapy and who is at greatest risk of toxicity is needed.

Survival of 32 months after diagnosis of metastatic GEJ adenocarcinoma in our patient suggests that multidisciplinary discussion and multimodality therapy in appropriately selected cases may result in longer survival.

## Figures and Tables

**Table 1:** Summary of the literature reviewed on neoadjuvant chemoradiotherapy in GEJ cancer.

	Median F/U (years)	Population (% of patients)	Randomization (patients)	Overall survival		Progression-free survival	
				Median (years)	3–5 years (%)	Median (years)	3–5 years (%)
Van Hagen et al. (2012) [4] CROSS trial	3.8	Resectable esophageal (73%) or GEJ (24%) CA	Carboplatin + paclitaxel/41.4 Gy + surgery (178)	4.1^*∗*^	47%^*∗*^ (5 yr)	—	—
			Surgery alone (188)	2.0^*∗*^	34%^*∗*^ (5 yr)	—	—

Tepper et al. (2008) [5] CALGB 9781 trial	6.0	Stages I–III of CA of esophagus or GEJ	Cisplatin + 5-FU/50.4 Gy + surgery (30)	4.5^*∗*^	39% (5 yr)	3.5^*∗*^	28% (5 yr)
			Surgery alone (26)	1.8^*∗*^	16% (5 yr)	1.0^*∗*^	15% (5 yr)

Stahl et al. (2009) [18] POET trial	3.8	Locally advanced adenocarcinoma of GEJ	Cisplatin + 5-FU + leucovorin/30 Gy + surgery (60)	2.8	47.4% (3 yr)	—	76.5% (3 yr)
			Cisplatin + 5-FU + leucovorin + surgery (59)	1.8	27.7% (3 yr)	—	59.0% (3 yr)

Burmeister et al. (2005) [19]	5.4	Esophageal CA including lower third/gastric cardia (79%)	Cisplatin + 5-FU/35 Gy + surgery (128)	1.9	11.7% (5 yr)	1.3	10.2% (5 yr)
			Surgery alone (128)	1.6	7.8% (5 yr)	1.0	7.0% (5 yr)

Walsh et al. (1996) [20]	0.8	Esophageal CA including lower third/cardia (85%)	Cisplatin + 5-FU/40 Gy + surgery (58)	1.3^*∗*^	32% (3 yr)^*∗*^	—	—
			Surgery alone (55)	0.9^*∗*^	6% (3 yr)^*∗*^	—	—

Urba et al. (2001) [21]	8.2	Stages I–III of CA of esophagus or GEJ	Cisplatin + 5-FU + vinblastine/45 Gy + surgery (50)	1.4	30% (3 yr)	—	28% (3 yr)
			Surgery alone (50)	1.5	16% (3 yr)	—	16% (3 yr)

(i) ^*∗*^Statistically significant value.

(ii) F/U: follow-up, CA: cancer, 5-FU: 5-fluorouracil, and yr: years.

(iii) —: not reported.
